# Evaluation of Nutritional Status of Intensive Care Unit COVID-19 Patients Based on the Nutritional Risk Screening 2002 Score

**DOI:** 10.1155/2022/2448161

**Published:** 2022-10-17

**Authors:** Sedigheh Ahmadi, Donya Firoozi, Mohammad Dehghani, Morteza Zare, Zeinab Mehrabi, Maryam Ghaseminasab-Parizi, Seyed Jalil Masoumi

**Affiliations:** ^1^Student Research Committee, Shiraz University of Medical Sciences, Shiraz, Iran; ^2^Department of Clinical Nutrition, School of Nutrition and Food Sciences, Shiraz University of Medical Sciences, Shiraz, Iran; ^3^Nutrition Research Center, School of Nutrition and Food Sciences, Shiraz University of Medical Sciences, Shiraz, Iran; ^4^Department of Internal Disaease, Shiraz University of Medical Sciences, Shiraz, Iran; ^5^Department of Health Education and Health Promotion, School of Health, Occupational Environment Research Center, Rafsanjan University of Medical Sciences, Rafsanjan, Iran; ^6^Gastroenterohepatology Research Center, Shiraz University of Medical Sciences, Shiraz, Iran; ^7^Center for Cohort Study of SUMS Employees' Health, Shiraz University of Medical Sciences, Shiraz, Iran

## Abstract

**Background:**

Patients with COVID-19 are susceptible to malnutrition, which is particularly concerning among critically ill patients. We evaluated the Nutritional Risk Screening 2002 (NRS-2002) score in such patients and determined its relationship with the hospitalization outcome.

**Methods:**

This cross-sectional study involved COVID-19 patients admitted to the intensive care units (ICUs) of Shahid Faghihi Hospital, Shiraz, Iran, between February and March 2021. We assessed the nutritional status using NRS-2002 and determined disease severity with the APACHE II index. Demographic information, weight, height, clinical signs, previous illness, medications, biochemical test results, and history of anorexia and weight loss were recorded. Data were analyzed using SPSS version 18.

**Results:**

The mean age of 100 patients was 55.36 ± 18.86 years. According to NRS-2002, 30%, 29%, and 41% of patients were at low risk, moderate risk, and high risk of malnutrition, respectively. Age and BUN increased significantly with NRS-2002, while albumin and hematocrit followed the opposite trend (*P* < 0.001). Patients who died had lower albumin and hematocrit levels but higher age, NRS-2002 scores, and BUN/creatinine levels than those who recovered. Multivariable logistic regression revealed that for every unit increase in the NRS-2002 score, the odds of mortality increased by 354% (OR: 4.54, CI: 1.48, 13.95, *P*=0.008).

**Conclusion:**

NRS-2002 is a valuable prognostic tool for critically ill COVID-19 patients, with each unit's rise in the score being associated with a 354% rise in the odds of mortality. Increased malnutrition risk was linked with higher age and BUN and lower albumin and hematocrit levels.

## 1. Introduction

Coronavirus disease 2019 (COVID-19) originated in China in late 2019, signaling the start of a global pandemic [1]. Typical symptoms include fever, cough, myalgia, and fatigue [2], and the disease can involve multiple organ systems [3]. Most patients are affected mildly and do not require hospitalization, but patients with severe disease may require mechanical ventilation and admission to an intensive care unit (ICU) [1]. While agents such as steroids can significantly reduce deaths in hospitalized COVID-19 patients [4], a drug of choice is yet to be established [5]. Hence, studies that improve disease understanding and management are still required.

Patients with COVID-19 are susceptible to malnutrition [6]. Dealing with fever, repairing damaged tissues, eradicating infection, and coping with disease complications augment the energy requirements of a patient with COVID-19. On the other hand, a decrease in food intake due to losing taste and appetite is also common, with diarrhea and gastrointestinal disorders sometimes experienced [6]. Malnutrition significantly affects the outcomes of patients with COVID-19; individuals with multiple comorbidities, older adults, and those with malnutrition are at increased risk of ICU admission and mortality [7]. ICU admission, particularly when prolonged, can result in malnutrition and muscle depletion, thereby increasing mortality and morbidity and impairing the quality of life in patients recovering from COVID-19 [8]. Therefore, improving the nutritional care of critically ill COVID-19 patients is vital, and efforts must be directed to identify and address malnutrition and prevent the associated adverse health outcomes. Hence, in this study, we evaluated the nutritional status among COVID-19 patients admitted to the ICU and determined the relationship between nutritional status and patient outcomes.

## 2. Patients and Methods

We conducted a cross-sectional investigation on patients with COVID-19 hospitalized in the ICUs of Shahid Faghihi Hospital, Shiraz, Iran, from February to March 2021. All ICU patients with COVID-19 diagnosed based on the reverse transcription-polymerase chain reaction (RT-PCR) test were selected. Pregnant and intubated patients, patients with parenteral nutrition, and patients with incomplete data were excluded. The sample size of 100 patients was determined by a statistical consultant using the formula for comparing means of continuous outcomes between two groups [9] based on a brief pilot study as well as a similar study [10]. The Ethics Committee of Shiraz University of Medical Sciences provided approval for the conduction of this study (code: IR.SUMS.REC.1399.1193).

### 2.1. Data Collection

In this study, we assessed nutritional status using the validated Nutritional Risk Screening 2002 (NRS-2002) scale [11–14]. This tool screens the risk of malnutrition using parameters such as the patient's age, body mass index (BMI), weight loss, calorie intake, and disease severity. An age of 70 or above receives 1 point, and nutritional impairment and disease severity are each scored from 0 to 3. The total score ranges from 0 to 7. A total score between 0 and 3 indicates a low risk of malnutrition; a score of 4 indicates moderate risk, while scores of 5 to 7 signify a high risk of malnutrition. Also, we assessed disease severity using the Acute Physiology and Chronic Health Evaluation II (APACHE II) index.

In the first 24 hours of ICU admission, patients' demographic information, weight, height, clinical signs, previous illness, medications, and history of anorexia and weight loss in the last three months were recorded through interviews with the patients or their caregivers. Patients' admission records were also used to complete the data. The amount of weight loss within three months was calculated from the difference between the patient's usual weight in the last three months and the current weight. Patients were weighed using a Seca scale (accurate to 0.1 kg); height was measured with 0.1 cm accuracy. By dividing the weight (kg) by height squared (m^2^), we calculated the body mass index (BMI). The daily calorie intake of patients was also recorded.

To calculate the APACHE II index, parameters including PaO_2_, rectal temperature, mean arterial pressure, blood pH, heart rate, respiratory rate, serum sodium, serum potassium, creatinine, hematocrit, white blood cell count, and Glasgow coma scale score were extracted from the patients' files. We also noted the on-file biochemical parameters, including creatinine, blood urea nitrogen (BUN), hematocrit, C-reactive protein (CRP), and albumin.

### 2.2. Statistical Analysis

We analyzed all data using SPSS version 18 (Chicago, IL, USA). Data distribution was investigated using the Kolmogorov–Smirnov statistical test. We used the mean and standard deviation to summarize continuous variables and frequency and percentage to summarize categorical variables. We used the independent *t*-test to compare quantitative factors when the distribution was normal, with the Mann–Whitney *U* test being used otherwise. We compared qualitative variables using the chi-squared test or Fisher's exact test as appropriate. We used ANOVA or the Kruskal–Wallis test to compare quantitative variables across multiple groups as appropriate, while the chi-squared test was used for qualitative variables. Univariate logistic regression models were used to predict the odds of malnutrition in the recovered and deceased patients. We minimized the impact of confounding variables by limiting our multivariable regression model to those variables with significance levels equal to or below 0.20. We computed the odds ratios (ORs) with their 95% confidence intervals (CIs). Statistical significance was signified by *P* values below 0.05.

## 3. Results

From February 19, 2021, to March 20, 2021, 134 patients from the ICU COVID-19 ward of Shahid Faghihi Hospital in Shiraz, Iran, were selected. After adjusting to the exclusion criteria, 34 patients were excluded from this study: 19 due to inaccessible dossiers, 7 due to incomplete anthropometric data, 2 due to transfer to other hospitals, 5 due to incomplete laboratory data, and 1 due to pregnancy. Ultimately, 100 patients (61 men and 39 women) were included in the final analysis.

In order to determine the factors associated with mortality, we compared various clinical parameters between patients with a hospitalization outcome of death or recovery (Table 1). The mean age of all patients was 55.36 ± 18.86 years; those who died were significantly older than those who recovered. Most patients were male (61%), and the length of hospitalization was 16.09 ± 13.65 and 19.52 ± 11.14 days in the recovered and deceased groups, respectively. The deceased group's average albumin (3.15 ± 0.55 vs. 3.52 ± 0.55 g/dl) and hematocrit levels (34.10 ± 7.03 vs. 38.67 ± 6.1 l/l) were significantly lower, but their BUN (30.51 ± 19.39 vs. 22.15 ± 13.12 mg/dl) and creatinine (1.70 ± 1.09 vs. 1.32 ± 0.98 mg/dl) levels were significantly higher (Table 1). According to NRS-2002, 30%, 29%, and 41% of patients were at low, moderate, and high risk of malnutrition, respectively. In the deceased group, most patients (70.5%) were at high risk of malnutrition; a significant difference was seen in NRS-2002 scores between the two groups (*P* < 0.001) (Table 1).

Table 2 compares the characteristics of the patients according to their NRS-2002 classification. Age (*P* < 0.001) and BUN (*P* < 0.001) increased significantly with NRS-2002, while albumin (*P* < 0.001) and hematocrit (*P* < 0.001) followed the opposite trend (Table 2).

A multivariable logistic regression model was fitted with variables compared between recovered and deceased patients (Table 3). For every unit increase in the NRS-2002 score, the odds of mortality with COVID-19 increased by 354% (OR: 4.54, CI: 1.48, 13.95, *P*=0.008). No significant correlation was seen between other parameters and odds of mortality (Table 3).

## 4. Discussion

The present study evaluated the nutritional status of patients with COVID-19 admitted in ICUs based on the NRS-2002 score and compared various measures between cases of recovery and mortality. The key findings were significantly greater NRS-2002 scores (i.e., increased risk of malnutrition) in those who were older, had higher BUN levels, or had lower albumin or hematocrit levels. Furthermore, a significantly larger number of patients who succumbed to COVID-19 were at high risk of malnutrition relative to those who survived; a 354% rise in the odds of mortality was seen for each unit rise in the NRS-2002 score.

Although certain treatments, such as steroids, can significantly reduce deaths in hospitalized COVID-19 patients [4], the lack of an established drug of choice [5] means that modifiable factors that affect patient outcomes must be identified and addressed. Our findings are a proof of concept and add to the growing body of evidence supporting the considerable impact of malnutrition on the outcomes of COVID-19 patients. Malnutrition, alongside increased age and comorbidities, is a risk factor for ICU admission and mortality due to COVID-19, making nutrition care essential [7]. As NRS-2002 acts as a strong and independent score to assess the risk of malnutrition-related mortality and adverse outcomes among hospitalized patients—a risk that can be modified by providing sufficient dietary support [11]. According to recent reviews, this screening tool is highly sensitive and comparable to alternative tools for identifying nutritional risk among COVID-19 patients [13, 14].

Age is a factor that is directly associated with the risk of mortality due to COVID-19 [15–18]. Malnutrition is highly prevalent in elderly patients and may partially account for this elevated mortality risk given its links with severe disease [6, 19, 20]. In the present study on critically ill COVID-19 patients, those who died had a greater mean age than those who recovered. This is in line with a similar study [21] and with the trend mentioned in the literature [5]. Interestingly, we found that those with a higher age also had a higher risk of malnutrition, as reported by Alikiaii et al. [21]. This is significant because the interaction between malnutrition and COVID-19 risk and severity seems to be bidirectional, with each able to affect the other [20, 22]. One study reviewed 4,187 hospitalized COVID-19 patients and revealed ten-fold higher odds of mortality among those who were malnourished relative to well-nourished individuals [19]. Another study on older, hospitalized COVID-19 patients also independently linked older age and higher NRS‐2002 scores with in‐hospital mortality [23].

Our study found that patients who achieved recovery had higher albumin levels than those who succumbed to COVID-19, as did the study of Alikiaii et al. [21]. High-quality evidence supports the idea that increased COVID-19 severity and a worsened prognosis are linked with hypoalbuminemia [24]. The mechanism behind this phenomenon remains elusive; however, an inflammatory process may be involved, where the permeation of albumin into the interstitial space is promoted, eventuating in hypoalbuminemia [24–26]. Hence, the independent role of albumin in predicting mortality in critically ill patients with COVID-19 declared by Li et al. may be explained by this mechanism [27]. While some authors [27, 28] have recommended that albumin be administered to such patients, the benefits of therapy are yet to be investigated.

Our study found that the risk of malnutrition was fairly balanced across the low (30%), moderate (29%), and high-risk (41%) categories overall. However, a significantly higher proportion of the patients who succumbed to COVID-19 were at high risk of malnutrition (NRS ≥5) relative to the patients who recovered. In fact, just over 70% of fatalities had a high risk of malnutrition. In contrast, one investigation found most (69.9%) ICU COVID-19 patients were at intermediate risk of malnutrition [21]. This difference may be explained by variations in socioeconomic status, diet, lifestyle, environmental conditions, viral load, and treatment timing across different study populations, as these factors can influence nutritional status [21, 29].

Researchers have previously highlighted the clinical utility of NRS-2002 in assessing nutritional risk and predicting the length of hospitalization in patients with COVID-19 [30]. In our study, the odds of mortality increased by 354% for each unit rise in the NRS-2002 score. This is in agreement with a similar study, which found that the NRS-2002 score correlated well with the disease mortality risk [21]. Moreover, a prospective multicenter study performed in 12 Argentinian ICUs revealed that an NRS-2002 score of 3 or above conferred a 2.25 [95% CI 1.01–5.01] times higher risk of COVID-19 mortality in the ICU [31]. Recent high-quality data indicate that by adversely affecting multiple organ systems, malnutrition leads to poor patient outcomes among COVID-19 patients [19]. A number of researchers have described the NRS index as useful and applicable in evaluating the prognosis of COVID-19 patients [14, 21, 32]. This is explained by the fact that this index takes into account factors including age, nutrition, comorbidities, and disease severity, with COVID-19 severity itself being predicted by poor nutritional status [33]. Hence, we seem to be able to predict the prognosis of COVID-19 patients using the NRS-2002 index, and a key strength of our study was the use of this strong [11], validated [12], and sensitive [13, 14] tool to acquire evidence regarding the impact of nutritional status on outcomes of ICU patients with COVID-19.

### 4.1. Limitations

The single-center, cross-sectional design of the study limits its ability to provide firm evidence. Furthermore, the relatively short study duration means that the possible confounding effects of time-dependent factors such as vaccination and the emergence of novel viral strains could not be evaluated. As such, we recommend continued collaborative research efforts on the national and international scale to produce high-quality evidence on this issue, as better patient outcomes can be achieved by devising appropriate strategies to identify and control nutritional risk in critically ill COVID-19 patients.

## 5. Conclusion

Among patients with COVID-19 admitted to the ICU, older patients and those with higher BUN levels or lower albumin or hematocrit levels were at increased risk of malnutrition according to the NRS-2002 scale, with each unit rise in this scale being associated with a 354% rise in the odds of mortality. Moreover, a significantly larger number of patients who succumbed to COVID-19 were at high risk of malnutrition compared with those who survived. These findings confirm that the NRS-2002 score is a valuable prognostic tool for patients with COVID-19 admitted to ICUs. Future studies should investigate the value of various interventions to modify the mortality risk among critically ill COVID-19 patients with higher NRS-2002 scores.

## Figures and Tables

**Table 1:** Characteristics of COVID-19 patients in the intensive care unit according to the hospitalization outcome, mean ± SD or N (%).

Variable	Overall (*n* = 100)	Recovered (*n* = 56)	Deceased (*n* = 44)	*P*-value
Age, years	55.36 ± 18.86	51.29 ± 18.74	60.82 ± 17.81	0.01
Gender, female	39 (39%)	18 (32.14%)	21 (47.7%)	
Body mass index, kg/m^2^	27.13 ± 6.04	27.88 ± 6.64	26.12 ± 5.01	0.14
NRS-2002				
Low risk	30 (30%)	27 (48.21%)	3 (6.8%)	<0.001
Moderate risk	29 (29%)	19 (33.92%)	10 (22.7%)	
High risk	41 (41%)	10 (17.85%)	31 (70.5%)	
Length of hospitalization, days		16.09 ± 13.65	19.52 ± 11.14	
Albumin, g/dl	3.36 ± 0.55	3.52 ± 0.55	3.15 ± 0.55	0.004
Hematocrit, l/l	36.68 ± 6.87	38.67 ± 6.1	34.10 ± 7.03	0.001
C-reactive protein, U/l	57.89 ± 20.09	54.04 ± 22.44	62.52 ± 15.90	0.07
Blood urea nitrogen, mg/dl	25.62 ± 16.45	22.15 ± 13.12	30.51 ± 19.39	0.02
Creatinine, mg/dl	1.76 ± 3.00	1.32 ± 0.98	1.70 ± 1.09	0.03

Independent sample *t*-test and Mann–Whitney *U* test for quantitative variables; chi-squared or Fisher's exact test for qualitative variables. NRS-2002: nutritional risk screening 2002.

**Table 2:** Characteristics of COVID-19 patients in the intensive care unit according to nutritional risk screening 2002 (NRS-2002) scores, mean ± SD or *N*.

Variable	Score ≤3 (*n* = 30)	Score = 4 (*n* = 29)	Score ≥5 (*n* = 41)	*P* value
Age, years	47.57 ± 16.67^†^	50.76 ± 18.44^§^	63.88 ± 17.69^§^	<0.001
Gender, female	12	9	15	0.76
Body mass index, kg/m^2^	28.85 ± 6.03	27.44 (6.17)	25.50 ± 5.65	0.13
C-reactive protein, U/L	51.76 ± 23.64	60.68 (14.94)	61.25 ± 19.26	0.30
Albumin, g/dl	3.73 ± 0.5^∗†^	3.30 (0.51)^∗^	3.10 ± 0.53^†^	<0.001
Hematocrit, L/L	39.71 ± 5.58^†^	38.60 ± 5.34^§^	32.91 ± 7.09^§^	<0.001
Blood urea nitrogen, mg/dL	17.79 ± 8.94^†^	20.67 ± 13.41^§^	35.11 ± 18.31^§^	<0.001
Creatinine, mg/dL	1.06 ± 0.18	1.26 ± 0.63	2.66 ± 4.61	0.06
Discharge status recovered	27	19	10	<0.001
Length of hospitalization, days	15.67 ± 16.22	18.18 ± 10.54	19.07 ± 11.33	0.05

ANOVA or Kruskal–Wallis test for quantitative variables; chi-squared test for qualitative variables. ^∗^A significant difference between scores 1 and 2; ^†^ between scores 1 and 3; ^§^between scores 2 and 3.

**Table 3:** Univariate and multivariate logistic model for the effects of nutritional risk screening 2002 (NRS-2002), age, and biochemical parameters on mortality.

Variable	Univariate model				Multivariate model			
	95% CI				95% CI			
	OR	Lower	Upper	*P* value	OR	Lower	Upper	*P* value
NRS-2002	3.99	2.26	7.05	<0.001	4.54	1.48	13.95	0.008
Age	1.02	1.006	1.05	0.01	1.04	0.98	1.09	0.15
Body mass index	0.95	0.88	1.01	0.15	0.97	0.80	1.17	0.75
C-reactive protein	1.02	0.99	1.04	0.06	1.008	0.96	1.05	0.70
Albumin	0.30	0.12	0.71	0.006	1.09	0.23	5.00	0.90
Hematocrit	0.89	0.83	0.96	0.003	0.94	0.82	1.08	0.45
Blood urea nitrogen	1.03	1.005	1.06	0.02	0.97	0.90	1.05	0.48
Creatinine	1.43	0.90	2.26	0.12	2.75	0.11	67.95	0.53

OR: odds ratio; CI: confidence interval.

## Data Availability

The data used to support the findings of this study are available from the corresponding author upon request.
